# New Modified Generalized Inverted Exponential Distribution and Its Applications

**DOI:** 10.3390/e28020161

**Published:** 2026-01-31

**Authors:** Zakeia A. Al-Saiary, Hana H. Al-Jammaz

**Affiliations:** 1 Department of Mathematics and Statistics, College of Science, University of Jeddah, Jeddah 22254, Saudi Arabia; 2200536@uj.edu.sa; 2 Department of Statistics and Operations Research, College of Science, Qassim University, Buraydah 51482, Saudi Arabia

**Keywords:** modified distribution, generalized inverted exponential, Rényi entropy, maximum likelihood estimation, Monte Carlo simulation

## Abstract

In this paper, a statistical model with three parameters is proposed which is called New Modified Generalized Inverted Exponential Distribution (MGIE). In addition, several statistical characteristics of the MGIE distribution are obtained, including quantile function, median, moments, mode, mean deviation, harmonic mean, reliability, hazard and odds functions and Rényi entropy. Moreover, the estimators of parameters are found using the maximum likelihood estimation method. A simulation study using the Monte Carlo method is performed to assess the behavior of the parameters. Finally, three real data sets are applied to demonstrate the importance of the proposed distribution.

## 1. Introduction

Given the data revolution and its importance in the modern era, and because traditional distributions cannot accurately represent the behavior of many data sets across various fields, statistical researchers have resorted to generat new distributions by creating families in order to provide greater flexibility and relevance. This highlights the importance of the widespread adoption of these families.

There are several examples of these families. A novel class of exponentiated generalized distributions was introduced by [1]. Type II power Topp–Leone-G family was proposed by [2]. The Lomax-G was generated by [3]. In 2020, a novel class of heavy-tailed distributions, which is referred to as a new extended family of heavy-tailed distribution, was found by [4]. A new Modified-X family was derived by [5]. The BurrXII-log-logistic family was introduced by [6]. In 2022, A new modified-G (NModi-G) family, utilizing a new statistical technique, was studied by [7]. Based on the previous group, we established our new modified model.

The cumulative distribution function (CDF) of the NModi-G family is given by

(1) $$G\left(x;\sigma ,\phi \right)=\frac{F(x;\phi )}{\sigma }\left[\sigma -1+F\left(x;\phi \right)\right],$$

where σ≥1,σ≤−1,x∈R, and F(x;ϕ) is a baseline CDF and ϕ represents the parameters of the baseline distribution.

The probability density function (PDF) of (1) is given by

(2) $$g\left(x;\sigma ,\phi \right)=\frac{f(x;\phi )}{\sigma }\left[\sigma -1+2F\left(x;\phi \right)\right].$$

Recently, many statisticians have shown interest in generalized inverted exponential distribution (GIED), which was introduced by [8]. For example, Topp–Leone generalized inverted exponential distribution (TLGIE) [9], type II Topp–Leone generalized inverted exponential distribution (TIITLGIE) [10]. Various approaches were discussed to find estimates of the shape and scale parameters by [11]. A new model of bivariate exponentiated generalized inverted exponential distribution was proposed by [12], and parameters were estimated when the data involved were censored samples of the first type by [13].

The GIED has the following PDF:

(3) $$f\left(x\right)=\left(\frac{\theta \lambda }{x^{2}}\right)\;e^{\frac{-\lambda }{x}}{\left[1-e^{\frac{-\lambda }{x}}\right]}^{\theta -1},\;x>0,\;\lambda ,\theta >0,$$

and the CDF is given by:

(4) $$F\left(x\right)=1-{\left[1-e^{\frac{-\lambda }{x}}\right]}^{\theta },\;x>0,\;\lambda ,\theta >0,$$

where θ is a shape parameter and λ is a scale parameter.

Although our model is based on the general framework of the NModi-G model family, it introduces specific modifications that were not found in previous work by depending on generalized inverted exponential distribution as a baseline. In particular, it provided better flexibility and suitability when we added the proposed distribution compared to other families, such as the exponential family and the Topp–Leone family. This is clearly demonstrated through applications on real data.

We chose the generalized inverted exponential (GIE) distribution as the baseline because of its diverse risk function shapes, which support a wide range of risk profiles. It also provides heavier tails and greater flexibility than the standard inverted exponential and demonstrated strong experimental performance in lifetime data modeling. These features make it a suitable and efficient baseline for developing the proposed model.

The rest of this paper is organized into the following sections. In Section 2, the PDF and CDF of MGIE will be presented. In addition, the major statistical properties of our model, such as the quantile function, moments, reliability function, hazard function, mean, median, and mode can be found in Section 3. Furthermore, Rényi entropy is derived in Section 4. In Section 5, we estimate the parameters of the model. Also, we perform a simulation study in Section 6. Finally, bio-sciences data sets are applied in Section 7. These data sets were primarily chosen because they are widely used in the evaluation of new statistical models and provide suitable data characteristics for testing the statistical performance of the proposed distribution. Also, the basic family from which the proposed distribution originated was designed to fit this type of data, as mentioned in [7].

## 2. The Modified Generalized Inverted Exponential Distribution

In this section, we introduce modified generalized inverted exponential distribution which contains three parameters (θ,λ,σ). The PDF and CDF of MGIE distribution is obtained by substituting Equations (3) and (4) in Equations (1) and (2):

(5) $$g\left(x\right)=\left(\frac{\theta \lambda }{x^{2}}\right)\;e^{\frac{-\lambda }{x}}{\left(1-e^{\frac{-\lambda }{x}}\right)}^{\theta -1}\left(1+\frac{1}{\sigma }-\frac{2}{\sigma }{\left(1-e^{\frac{-\lambda }{x}}\right)}^{\theta }\right),$$

and

(6) $$G\left(x\right)=1-{\left(1-e^{\frac{-\lambda }{x}}\right)}^{\theta }\left(\frac{1}{\sigma }+1-\frac{1}{\sigma }{\left(1-e^{\frac{-\lambda }{x}}\right)}^{\theta }\right),$$

where θ is a shape parameter and λ,σ are scale parameters.

Using the following series:

(1−x)n=∑i=0nni(−1)i(x)i(1)n−i,

we can reformat PDF and CDF as follows:

(7) $$g\left(x\right)=\psi_{1}{\left(x\right)}^{-2}e^{\frac{-\lambda }{x}\left(i+1\right)}+\psi_{2}{\left(x\right)}^{-2}e^{\frac{-\lambda }{x}\left(j+1\right)},$$

where

(8) ψ1(i,θ,λ,σ)=(1+1σ)(θλ)∑i=0∞θ−1i(−1)i,

(9) ψ2(j,θ,λ,σ)=(−2σ)(θλ)∑j=0∞2θ−1j(−1)j,

and

(10) $$G\left(x\right)=1-\left[\zeta_{1}\;e^{\frac{-\lambda }{x}i}-\zeta_{2}\;e^{\frac{-\lambda }{x}j}\right],$$

where

(11) ζ1(i,θ,σ)=(1σ+1)∑i=0∞θi(−1)i,

(12) ζ2(j,θ,σ)=(1σ)∑j=0∞2θj(−1)j.

These formulas that involve infinite series allow for an accurate representation of the theoretical properties of the proposed distribution and are also common in the literature on generated distributions. From a practical standpoint, these expressions can be efficiently evaluated numerically using available statistical packages like R and Mathematica.

### Some Ideal Sub Models as Special Cases from the Proposed Distribution

- For θ=1, the proposed distribution in (5) transforms to Modified Inverse Exponential distribution (MIE) and it is given as follows:
  (13) $$g\left(x\right)=\frac{\lambda }{x^{2}}\;e^{\frac{-\lambda }{x}}\left(1-\frac{1}{\sigma }+\frac{2}{\sigma }e^{\frac{-\lambda }{x}}\right).$$
- For θ=1 and λ=1, the proposed distribution transforms to Modified Standard Inverse Exponential distribution (MSIE) and it is given as
  (14) $$g\left(x\right)=\frac{1}{x^{2}}e^{\frac{-1}{x}}\left(1-\frac{1}{\sigma }+\frac{2}{\sigma }\;e^{\frac{-1}{x}}\right).$$
- For λ=1, the proposed distribution transforms to Modified Generalized Standard Inverse Exponential distribution (MGSIE) and it is given as
  (15) $$g\left(x\right)=\frac{\theta }{x^{2}}\;e^{\frac{-1}{x}}{\left(1-e^{\frac{-1}{x}}\right)}^{\theta -1}\left(1+\frac{1}{\sigma }-\frac{2}{\sigma }{\left(1-e^{\frac{-1}{x}}\right)}^{\theta }\right).$$
- For σ=∞, the proposed distribution transforms to Generalized Inverted Exponential distribution (GIED) and it is given as
  (16) $$g\left(x\right)=\left(\frac{\theta \lambda }{x^{2}}\right)\;e^{\frac{-\lambda }{x}}{\left(1-e^{\frac{-\lambda }{x}}\right)}^{\theta -1}.$$

**Figure 1:**
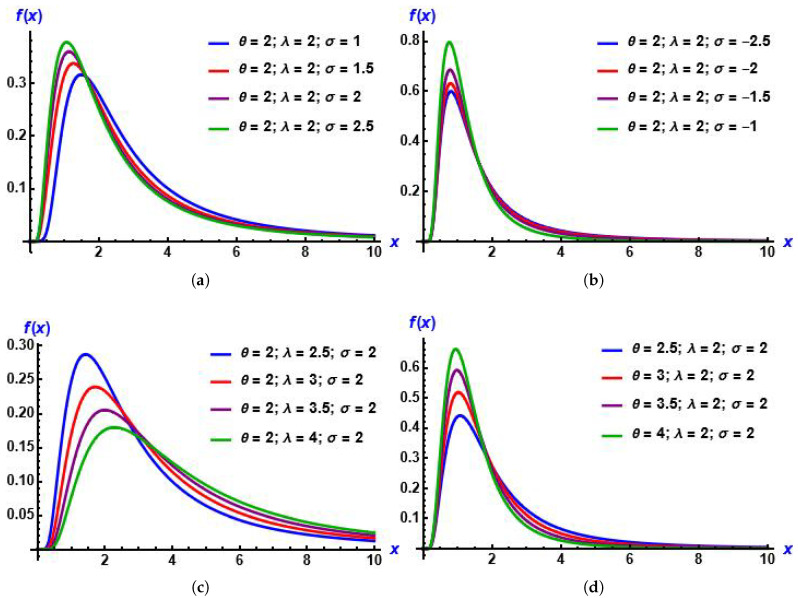
Plots of the PDF of the MGIE distribution for diverse values of the parameters. (**a**) σ increases; (**b**) σ decreases; (**c**) λ increases; (**d**) θ increases.

**Figure 2:**
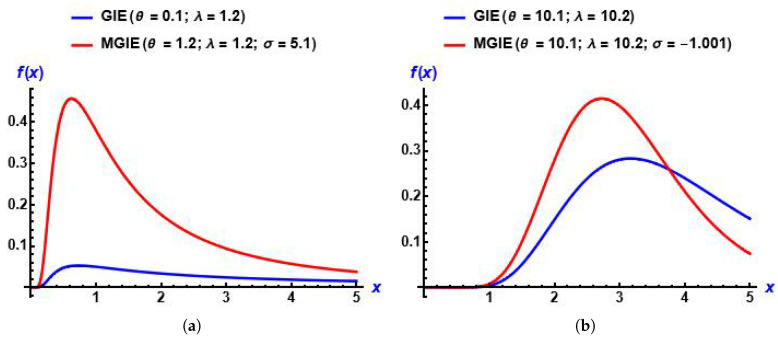
Plots of the PDF of the GIED and MGIE distributions for diverse values of the parameters. (**a**) σ is positive; (**b**) σ is negative.

From plots in Figure 1, we can see that the MGIE distribution is skewed to the right and unimodal. Also, Figure 2 shows the effect of the added parameter on the baseline distribution.

## 3. Properties of MGIE Distribution

### 3.1. Quantile and Median

We can get the quantile function as follows:

(17) $$x=Q\left(u\right)=G^{-1}\left(u\right)=F^{-1}\left(k\right),$$

where *k* is the solution of $\left(\sigma -1\right)k+k^{2}-\sigma u$. See [5]

The quantile function of the MGIE distribution is given by

(18) $$Q\left(u\right)=\frac{-\lambda }{Log[\left(1-{\left(1-\frac{1}{2}\left(1-\sigma +\sqrt{1-2\;\sigma +4\;u\;\sigma +\sigma^{2}}\right)\right)}^{\frac{1}{\theta }}\right)]}.$$

By putting U = 0.5 in the previous equation, we deduce the median of the MGIE distribution as

$$m=\frac{-\lambda }{Log[\left(1-{\left(1-\frac{1}{2}\left(1-\sigma +\sqrt{1-2\;\sigma +4\;\left(0.5\right)\;\sigma +\sigma^{2}}\right)\right)}^{\frac{1}{\theta }}\right)]}.$$

### 3.2. The $r^{th}$ Moment

To find the $r^{th}$ moment for x which has the MGIE distribution, we use the following equation:

$$\overset{´}{\mu_{r}}=E\left(x^{r}\right)=\int_{-\infty }^{\infty }x^{r}g\left(x\right)dx,$$

by substituting Equation (7) in the previous equation:

(19) $$\overset{´}{\mu_{r}}=\int_{0}^{\infty }x^{r}\left[\psi_{1}{\left(x\right)}^{-2}e^{\frac{-\lambda }{x}\left(i+1\right)}+\psi_{2}{\left(x\right)}^{-2}e^{\frac{-\lambda }{x}\left(j+1\right)}\right]dx,$$

where $\psi_{1}$, $\psi_{2}$ are given in Equations (8) and (9), respectively.

Let

$$u=\frac{\lambda }{x}\left(i+1\right),\;w=\frac{\lambda }{x}\left(j+1\right),$$

therefore, we get the $r^{th}$ Moment of the MGIE distribution:

(20) $$\begin{array}{cc} \overset{´}{\mu_{r}} & =[\left(\psi_{1}{\left(\lambda \right)}^{r-1}{\left(i+1\right)}^{r-1}\left(\sum_{n=0}^{\infty }\frac{{\left(-1\right)}^{n}}{(n-r+1)n!}+E_{r}\left(1\right)\right)\right]\\ \; & +[\left(\psi_{2}{\left(\lambda \right)}^{r-1}{\left(j+1\right)}^{r-1}\left(\sum_{n=0}^{\infty }\frac{{\left(-1\right)}^{n}}{(n-r+1)n!}+E_{r}\left(1\right)\right)\right],\;n>r-1, \end{array}$$

note that the $E_{r}\left(1\right)$ is generalized exponential integral [9]. It is given by

$$E_{n}\left(z\right)=\int_{1}^{\infty }\frac{e^{-zt}}{t^{n}}dt,$$

and $\psi_{1}$, $\psi_{2}$ are given in Equations (8) and (9), respectively.

Furthermore, we can find the mean of the MGIE distribution by equating *r* to 1:

(21) $$\mu =[\left(\psi_{1}\left(\sum_{n=0}^{\infty }\frac{{\left(-1\right)}^{n}}{n\;n!}+E_{1}\left(1\right)\right)\right]+[\left(\psi_{2}\left(\sum_{n=0}^{\infty }\frac{{\left(-1\right)}^{n}}{n\;n!}+E_{1}\left(1\right)\right)\right],$$

by substituting Equation (20) in the following formula:

$$M_{x}\left(t\right)=\sum_{r=0}^{\infty }\frac{t^{r}}{r!}E\left(x^{r}\right),$$

we obtain the moment generating function (MGF) of the MGIE distribution:

$$\begin{array}{cc} M_{x}\left(t\right) & =\sum_{r=0}^{\infty }\frac{t^{r}}{r!}[\left(\psi_{1}{\left(\lambda \right)}^{r-1}{\left(i+1\right)}^{r-1}\left(\sum_{n=0}^{\infty }\frac{{\left(-1\right)}^{n}}{(n-r+1)n!}+E_{r}\left(1\right)\right)\right]\\ \; & +[\left(\psi_{2}{\left(\lambda \right)}^{r-1}{\left(j+1\right)}^{r-1}\left(\sum_{n=0}^{\infty }\frac{{\left(-1\right)}^{n}}{(n-r+1)n!}+E_{r}\left(1\right)\right)\right],\;n>r-1. \end{array}$$

### 3.3. Skewness and Kurtosis

The skewness and kurtosis are important statistical measures. Bowley’s coefficient of skewness is also known as quartile skewness [14] and it is given by:

(22) $$B=\frac{Q\left(\frac{3}{4}\right)-2Q\left(\frac{1}{2}\right)+Q\left(\frac{1}{4}\right)}{Q\left(\frac{3}{4}\right)-Q\left(\frac{1}{4}\right)}.$$

We can derive Moors’ coefficient of kurtosis by using octileis [15], which separate the dataset into evenly sized divisions. It has the following formula:

(23) $$M=\frac{Q\left(\frac{7}{8}\right)-Q\left(\frac{5}{8}\right)+Q\left(\frac{3}{8}\right)-Q\left(\frac{1}{8}\right)}{Q\left(\frac{6}{8}\right)-Q\left(\frac{2}{8}\right)},$$

where *Q*(.) is given in (18).

### 3.4. Reliability Function

The reliability function is familiar as a survival function. It is used to determine the probability that the object of our interest will not fail over time. It is given by:

(24) R(x)=1−G(x),

Therefore, the reliability function for the MGIE distribution can be derived by substituting Equation (6) in Equation (24):

(25) $$R\left(x\right)=\tau (\left(\frac{1}{\sigma }+1\right)-\frac{1}{\sigma }\tau ),$$

where

(26) $$\tau \left(x;\theta ,\lambda \right)={\left(1-e^{\frac{-\lambda }{x}}\right)}^{\theta }.$$

**Figure 3:**
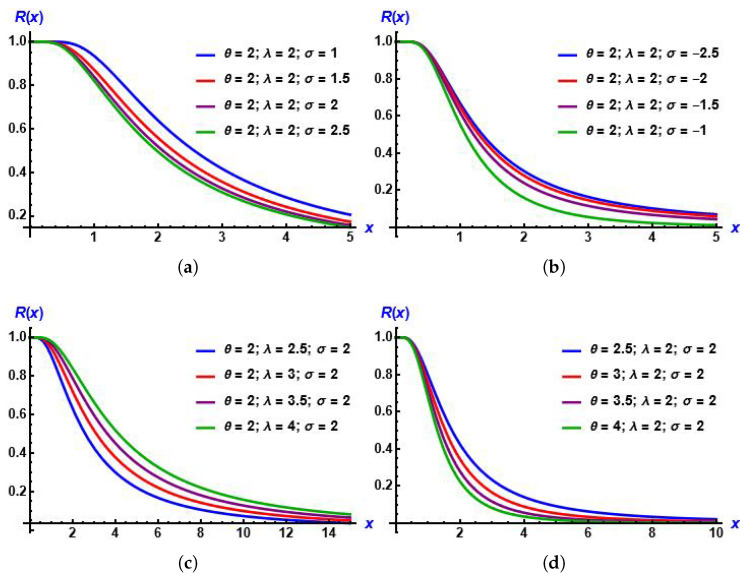
Plots of reliability function of the MGIE distribution for diverse values of the parameters. (**a**) σ increases; (**b**) σ decreases; (**c**) λ increases; (**d**) θ increases.

From plots in Figure 3, it is obvious that for any value of all parameters, the reliability function of the MGIE distribution decreases.

### 3.5. Hazard Function

The hazard function characterizes failure at a given time x, supposing that the object has survived up to that time. It is defined mathematically as

(27) $$h\left(x\right)=\frac{g(x)}{1-G(x)},$$

Thus, the hazard function for the MGIE distribution can be obtained by substituting Equations (5) and (6) in Equation (27):

(28) $$h\left(x\right)=\frac{\theta \lambda x^{-2}e^{\frac{-\lambda }{x}}\left(1+\frac{1}{\sigma }-\frac{2}{\sigma }{\left(1-e^{\frac{-\lambda }{x}}\right)}^{\theta }\right)}{\left(1-e^{\frac{-\lambda }{x}}\right)\left(\frac{1}{\sigma }+1-\frac{1}{\sigma }{\left(1-e^{\frac{-\lambda }{x}}\right)}^{\theta }\right)}.$$

**Figure 4:**
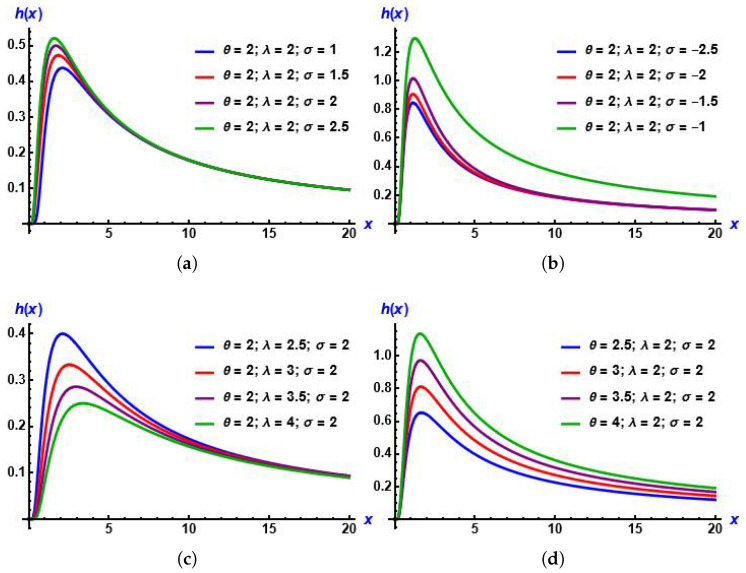
Plots of hazard function of the MGIE distribution for diverse values of the parameters. (**a**) σ increases; (**b**) σ decreases; (**c**) λ increases; (**d**) θ increases.

**Figure 5:**
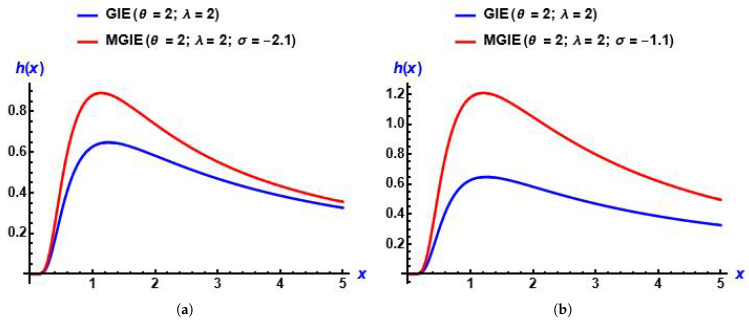
Plots of hazard function of the GIED and MGIE distributions for diverse parameter values. (**a**) σ is negative; (**b**) σ is negative.

From plots in Figure 4, we can recognize that from several values of parameters, the curves of the hazard function are upside-down bathtub-shaped. Also, they increase then decrease, which is advantageous in survival analysis. In addition, Figure 5 shows the effect of the added parameter on the baseline distribution.

### 3.6. Mode

The mode can be found by deriving the PDF of the MGIE distribution with respect to *x* as follows:

$$\frac{dg(x)}{dx}=0,$$

By substituting Equation (5) in the previous equation, we obtain

$$\begin{array}{cc} \; & f\left(x\right)[\frac{-2}{x}+\frac{\lambda }{x^{2}}-\left(\frac{\lambda }{x^{2}}\left(\theta -1\right)\;e^{\frac{-\lambda }{x}}{\left(1-e^{\frac{-\lambda }{x}}\right)}^{-1}\right)+\frac{2\theta \lambda }{\sigma x^{2}}\;e^{\frac{-\lambda }{x}}{\left(1-e^{\frac{-\lambda }{x}}\right)}^{\theta -1}\\ \; & {\left(1+\frac{1}{\sigma }-\frac{2}{\sigma }{\left(1-e^{\frac{-\lambda }{x}}\right)}^{\theta }\right)}^{-1}]=0, \end{array}$$

which gives

(29) $$\begin{array}{cc} \; & \frac{-2}{x}+\frac{\lambda }{x^{2}}-\left(\frac{\lambda }{x^{2}}\left(\theta -1\right)\;e^{\frac{-\lambda }{x}}{\left(1-e^{\frac{-\lambda }{x}}\right)}^{-1}\right)+\frac{2\theta \lambda }{\sigma x^{2}}\;e^{\frac{-\lambda }{x}}{\left(1-e^{\frac{-\lambda }{x}}\right)}^{\theta -1}\\ \; & {\left(1+\frac{1}{\sigma }-\frac{2}{\sigma }{\left(1-e^{\frac{-\lambda }{x}}\right)}^{\theta }\right)}^{-1}=0. \end{array}$$

As we can see, it is a non-linear equation. Therefore, we will use the Newton–Raphson method to solve this equation.

### 3.7. Odds Function

The odds function describes the ratio of an event to its non-occurrence. It is given by

$$O\left(x\right)=\frac{G(x)}{R(x)},$$

by substituting Equations (6) and (25) with the previous equation:

(30) $$O\left(x\right)=\frac{1-[\zeta_{1}\;e^{\frac{-\lambda }{x}i}-\zeta_{2}\;e^{\frac{-\lambda }{x}j}]}{\tau (\left(\frac{1}{\sigma }+1\right)-\frac{1}{\sigma }\tau )},$$

where $\zeta_{1}$, $\zeta_{2}$ and τ are given in Equations (11), (12) and (26), respectively.

**Figure 6:**
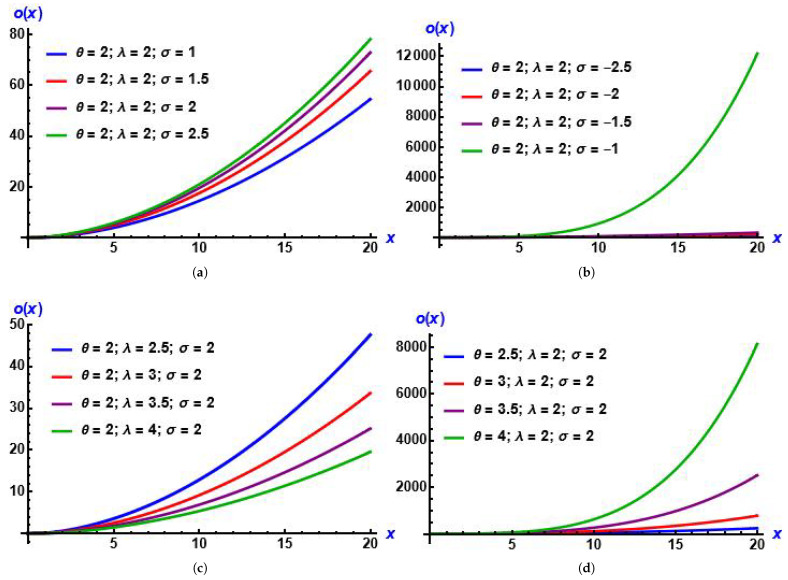
Plots of odds function of the MGIE distribution for diverse values of the parameters. (**a**) σ increases; (**b**) σ decreases; (**c**) λ increases; (**d**) θ increases.

From Figure 6, we find that for various values of parameters, the odd function increases.

### 3.8. Harmonic Mean

If we have the adverse of a set of values $x_{1},x_{2},\ldots ,x_{n}$, the inverse of its arithmetic mean is known as the harmonic mean. It is defined by

(31) $$H_{m}\left(x\right)=\frac{1}{E(\frac{1}{x})}=\frac{1}{\int_{0}^{\infty }x^{-1}g\left(x\right)dx},$$

let

$$I=\int_{0}^{\infty }x^{-1}g\left(x\right)dx,$$

to derive the harmonic mean of the MGIE distribution, we use Equation (7) in the previous equation:

$$I=\psi_{1}\int_{0}^{\infty }x^{-3}e^{\frac{-\lambda }{x}\left(i+1\right)}dx+\psi_{2}\int_{0}^{\infty }x^{-3}e^{\frac{-\lambda }{x}\left(j+1\right)}dx,$$

let

$$u=\frac{\lambda }{x}\left(i+1\right),w=\frac{\lambda }{x}\left(j+1\right),$$

Thus, we get the harmonic mean of the MGIE distribution:

(32) $$H_{m}\left(x\right)={\left[\frac{1}{\lambda^{2}}\left(\frac{\psi_{1}}{{\left(i+1\right)}^{2}}+\frac{\psi_{2}}{{\left(j+1\right)}^{2}}\right)\right]}^{-1}.$$

From Table 1, it is obvious that the alteration values of parameters of the MGIE distribution influence the behavior of its properties. Moreover, the MGIE distribution is unimodal and skewed to the right because, for any parameter values, the mode is less than the median and the median is less than mean. It is clear that if θ and σ increase, the mean, median, mode, harmonic mean, skewness, and kurtosis decrease. If λ increases, the mean, median, mode, and harmonic mean decrease, while the values of skewness and kurtosis do not change.

### 3.9. The Mean Deviation and the Median Deviation

The mean deviation and the median deviation are measures of dispersion that present data deviation from the mean and median. They can be found as follows:

#### 3.9.1. The Mean Deviation About the Mean

We can derive the mean deviation about the mean as follows:

(33) $$\begin{array}{cc} D(\mu ) & =E\left|x-\mu \right|\\ \; & =\int_{0}^{\infty }\left|x-\mu \right|\;g\left(x\right)dx. \end{array}$$

To derive the mean deviation of the MGIE distribution, substitute Equation (10) in Equation (33); then, we get

(34) $$\begin{array}{cc} D(\mu ) & =2\int_{0}^{\mu }\left[1-\left(\zeta_{1}\;e^{\frac{-\lambda }{x}i}-\zeta_{2}\;e^{\frac{-\lambda }{x}j}\right)\right]dx\\ \; & =2[\mu -\left(\zeta_{1}\left(\frac{\mu^{2}}{\lambda i}\;e^{\frac{-\lambda }{\mu }i}\right)-\zeta_{2}\left(\frac{\mu^{2}}{\lambda j}\;e^{\frac{-\lambda }{\mu }j}\right)\right)], \end{array}$$

where $\zeta_{1}$ and $\zeta_{2}$ are given in Equations (11) and (12) respectively.

#### 3.9.2. The Mean Deviation About the Median

We can derive the mean deviation about the median as follows:

(35) $$\begin{array}{cc} D(m) & =E\left|x-m\right|\\ \; & =\int_{0}^{\infty }\left|x-m\right|\;g\left(x\right)dx, \end{array}$$

To derive the median deviation of the MGIE distribution, substitute Equation (10) in Equation (35); then, we get

(36) $$\begin{array}{cc} D(m) & =\mu -m+2\int_{0}^{m}\left[1-\left(\zeta_{1}\;e^{\frac{-\lambda }{x}i}-\zeta_{2}\;e^{\frac{-\lambda }{x}j}\right)\right]dx\\ \; & =\mu -m+2[m-\left(\zeta_{1}\left(\frac{m^{2}}{\lambda i}\;e^{\frac{-\lambda }{m}i}\right)-\zeta_{2}\left(\frac{m^{2}}{\lambda j}\;e^{\frac{-\lambda }{m}j}\right)\right)], \end{array}$$

where $\zeta_{1}$ and $\zeta_{2}$ are given in Equations (11) and (12), respectively.

### 3.10. Order Statistics

For a random sample $x_{1},x_{2},\ldots ,x_{n}$ have the MGIE distribution with PDF g(x) and CDf G(x). The order statistics show that we should arrange these values in ascending order $x_{1}\leq x_{2}\leq \ldots \leq x_{n}$, and then the PDF of $x_{j}$ is defined as

(37) $$g\left(x_{j}\right)=\frac{n!}{(j-1)!\;(n-j)!}\;g\left(x\right)\;G{\left(x\right)}^{j-1}\;{\left[1-G\left(x\right)\right]}^{n-j},$$

By substituting Equations (5) and (6) in the previous equation, we get the PDF of the order statistic $x_{j}$:

$$\begin{array}{cc} g(x_{j}) & =\frac{n!}{(j-1)!\;(n-j)!}\;\frac{\theta \lambda }{x_{j}^{2}}\;e^{\frac{-\lambda }{x_{j}}}{\left(1-e^{\frac{-\lambda }{x_{j}}}\right)}^{\theta -1}\left[1+\frac{1}{\sigma }-\frac{2}{\sigma }{\left(1-e^{\frac{-\lambda }{x_{j}}}\right)}^{\theta }\right]\\ \; & \times {\left[1-{\left(1-e^{\frac{-\lambda }{x_{j}}}\right)}^{\theta }\left(\frac{1}{\sigma }+1-\frac{1}{\sigma }{\left(1-e^{\frac{-\lambda }{x_{j}}}\right)}^{\theta }\right)\right]}^{j-1}\\ \; & \times {\left[1-\left[1-{\left(1-e^{\frac{-\lambda }{x_{j}}}\right)}^{\theta }\left(\frac{1}{\sigma }+1-\frac{1}{\sigma }{\left(1-e^{\frac{-\lambda }{x_{j}}}\right)}^{\theta }\right)\right]\right]}^{n-j},\;x_{j}>0. \end{array}$$

We can find the PDF of the largest order statistic by using this formula:

(38) $$g\left(x_{n}\right)=n\;g\left(x\right)\;{\left[G\left(x\right)\right]}^{n-1},$$

thus

$$\begin{array}{cc} g(x_{n}) & =\frac{n\theta \lambda }{x_{n}^{2}}\;e^{\frac{-\lambda }{x_{n}}}{\left(1-e^{\frac{-\lambda }{x_{n}}}\right)}^{\theta -1}\left[1+\frac{1}{\sigma }-\frac{2}{\sigma }{\left(1-e^{\frac{-\lambda }{x_{n}}}\right)}^{\theta }\right]\\ \; & \times {\left[1-{\left(1-e^{\frac{-\lambda }{x_{n}}}\right)}^{\theta }\left(\frac{1}{\sigma }+1-\frac{1}{\sigma }{\left(1-e^{\frac{-\lambda }{x_{n}}}\right)}^{\theta }\right)\right]}^{n-1},\;x_{n}>0. \end{array}$$

The smallest order statistic is given by using the following formula:

(39) $$g\left(x_{1}\right)=n\;g\left(x\right)\;{\left[1-G\left(x\right)\right]}^{n-1},$$

thus

$$\begin{array}{cc} g(x_{1}) & =\frac{n\theta \lambda }{x_{1}^{2}}e^{\frac{-\lambda }{x_{1}}}{\left(1-e^{\frac{-\lambda }{x_{1}}}\right)}^{\theta -1}\left[1+\frac{1}{\sigma }-\frac{2}{\sigma }{\left(1-e^{\frac{-\lambda }{x_{1}}}\right)}^{\theta }\right]\\ \; & \times {\left[1-\left[1-{\left(1-e^{\frac{-\lambda }{x_{1}}}\right)}^{\theta }\left(\frac{1}{\sigma }+1-\frac{1}{\sigma }{\left(1-e^{\frac{-\lambda }{x_{1}}}\right)}^{\theta }\right)\right]\right]}^{n-1},\;x_{1}>0. \end{array}$$

## 4. Rényi Entropy of the MGIE Distribution

Rényi entropy was introduced by [16]. It is considered to be one of the generalizations of Shannon’s entropy [17]. Entropy is used in many fields like physics and engineering. In physics, entropy is defined as the second law of thermodynamics. A thermodynamic system is considered to be a measure of the system’s disorder and that varies directly with any reversible change in heat in the system and inversely with the temperature of the system. In statistical mechanics, entropy is a measure of uncertainty, which remains relevant to a system after its macroscopic properties, such as temperature, pressure, and volume, have been observed.

**Theorem** **1.** *The Rényi entropy of MGIE is given by*

(40) Rδ(x)=11−δ×log[λ(θλ)δ∑i=0n∑j=0nδi(θ−1)δ+θij(−1)i+j(2σ)i(1+1σ)δ−i(Γ(δ+1)(δ+j)δ+1)],

The proof is given in Appendix A.1.

## 5. Parameters Estimation

### 5.1. Maximum Likelihood Estimation

This subsection extracts the maximum likelihood estimation, which is used to find estimators of parameters. Let $x_{1},x_{2},\ldots ,x_{n}$ be a random sample from the MGIE distribution, then we can define the likelihood function by

$$L=\prod_{i=1}^{n}g\left(x_{i}\right),$$

The log-likelihood function is defined by

$$\begin{array}{c} L=\frac{{\left(\theta \lambda \right)}^{n}}{\prod_{i=1}^{n}{\left(x_{i}\right)}^{2}}\times e^{-\sum_{i=1}^{n}\frac{\lambda }{x_{i}}}\times \prod_{i=1}^{n}{\left(1-e^{\frac{-\lambda }{x_{i}}}\right)}^{\theta -1}\times \prod_{i=1}^{n}\left(1+\frac{1}{\sigma }-\frac{2}{\sigma }{\left(1-e^{\frac{-\lambda }{x_{i}}}\right)}^{\theta }\right), \end{array}$$

(41) $$\begin{array}{cc} l=\log L & =n\log\left(\theta \right)+n\log\left(\lambda \right)-2\sum_{i=1}^{n}\log\left(x_{i}\right)-\sum_{i=1}^{n}\frac{\lambda }{x_{i}}+\left(\theta -1\right)\sum_{i=1}^{n}\log\left(1-e^{\frac{-\lambda }{x_{i}}}\right)\\ \; & +\sum_{i=1}^{n}\log\left(1+\frac{1}{\sigma }-\frac{2}{\sigma }{\left(1-e^{\frac{-\lambda }{x_{i}}}\right)}^{\theta }\right), \end{array}$$

Differentiate the previous Equation (*l*) with respect to the parameters of our model θ, λ and σ, respectively.

All differentials are given in Appendix A.2.

### 5.2. Fisher Information

The Fisher method is used to obtain information for an unknown parameter by measuring another random, measurable variable.

Let $\underline{\varepsilon }=\left(\hat{\theta },\hat{\lambda },\hat{\sigma }\right)$ is a community of parameters for the MGIE distribution. Then, the Fisher information matrix is given as

$${\hat{I}}_{n}^{-1}\left(\underline{\hat{\varepsilon }}\right)=\left[\begin{array}{ccc} var(\hat{\theta }) & cov(\hat{\theta },\hat{\lambda }) & cov(\hat{\theta },\hat{\sigma })\\ cov(\hat{\lambda },\hat{\theta }) & var(\hat{\lambda }) & cov(\hat{\lambda },\hat{\sigma })\\ cov(\hat{\sigma },\hat{\theta }) & cov(\hat{\sigma },\hat{\lambda }) & var(\hat{\sigma }) \end{array}\right],$$

$${\hat{I}}_{n}^{-1}\left(\underline{\hat{\varepsilon }}\right)={\left(-\left(\frac{\partial^{2}logl}{\partial \varepsilon_{i}\partial \varepsilon_{j}}\right)\right)}_{\varepsilon =\hat{\varepsilon }},$$

To find this matrix, we need to find the second partial differentials of maximum likelihood estimation for all the parameters related to the distribution.

All differentials are given in Appendix A.3.

## 6. Simulation Study

To assess the efficiency of maximum likelihood estimators, in this section, we will find the estimates of the distribution parameters using the Mathematica (13.0) program and conduct simulations for different sample sizes, n = 10, 30, 50, 100, 150 and 200 from MGIE distribution. We will repeat the simulation N = 1000 and provide the maximum likelihood estimate (MLE), bias, mean squared error (MSE) and standard error (SE).

From Table 2 and Table 3, it is obvious that the maximum likelihood estimator is efficient and consistent, as we find that the MLE values approach the initial parameter with increasing sample size. In addition, we note that the bias, MSE, and SE become as low as possible.

## 7. Application

We will show the importance of the MGIE distribution by applying three real data points from bio-sciences fields. A method was used to estimate unknown parameters for the maximum likelihood estimation. To calculate this, we used Mathematica (13.0). We compared the suitability of the proposed distribution, which is the MGIE distribution with TIITLGIE distribution, exponentiated generalized inverted exponential distribution (EGIE) [18], Log-normal distribution [19], and Weibull distribution [20].

The comparison was performed according to the following criteria, so that the best distribution was the one with a lower value than the other distributions. The criteria were the Akaike information criterion (AIC) [21], Bayesian information criterion (BIC) [22], Corrected Akaike information criteria (CAIC) [23] and Hannan–Quinn information criterion (HQIC) [24].

### 7.1. Data Set 1

In this subsection, we show data from Saudi Arabia which consists of the number of deaths due to COVID-19 on 83 days, from 30 May to 20 August [25]: 17, 22, 23, 22, 24, 30, 32, 32, 34, 36, 34, 37, 36, 38, 36, 39, 40, 39, 41, 39, 48, 45, 46, 37, 40, 39, 41, 41, 46, 37, 40, 48, 50, 49, 54, 50, 56, 58, 52, 49, 42, 41, 51, 30, 42, 20, 40, 42, 45, 37, 40, 39, 37, 34, 44, 34, 37, 31, 30, 27, 29, 27, 26, 24, 21, 30, 32, 35, 36, 35, 38, 37, 37, 32, 34, 36, 34, 35, 31, 39, 28, 34, 36.

We will calculate the descriptive statistics for this data as shown in the Table 4.

**Figure 7:**
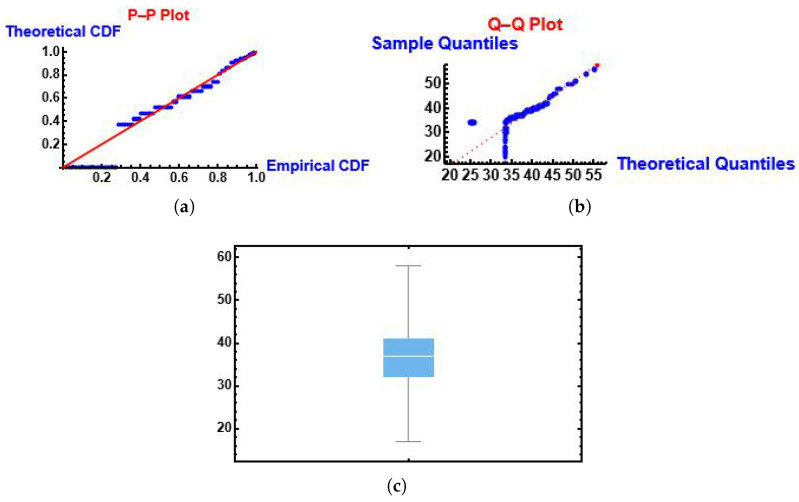
PP, QQ and box plots of the MGIE distribution using the number of deaths due to COVID-19 data. (**a**) PP plot; (**b**) QQ plot; (**c**) box plot.

From Figure 7 we can see that the MGIE distribution follows the fitted PP (probability-probability) and QQ (quantile-quantile) functions. Also, we can see the box plot.

**Figure 8:**
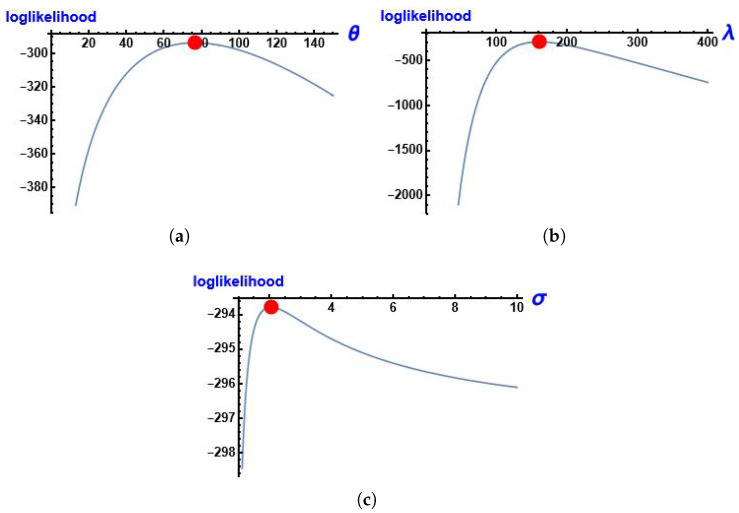
The profile of the log-likelihood function of the parameters using data about the number of deaths due to COVID-19. (**a**) log-likelihood function of θ; (**b**) log-likelihood function of λ; (**c**) log-likelihood function of σ.

Figure 8 shows the log-likelihood function of the parameters of MGIE distribution θ, λ and σ using this data.

Table 5 presents the Kolmogorov-Smirnov test to determine the goodness of fit and the *p*-value for MGIE distribution and competing models.

From Table 6 we note that the MGIE distribution consistently outperforms competitive distributions in all the criteria used for the study. This gives the MGIE distribution practically significant and supports its use as a flexible and effective alternative.

**Figure 9:**
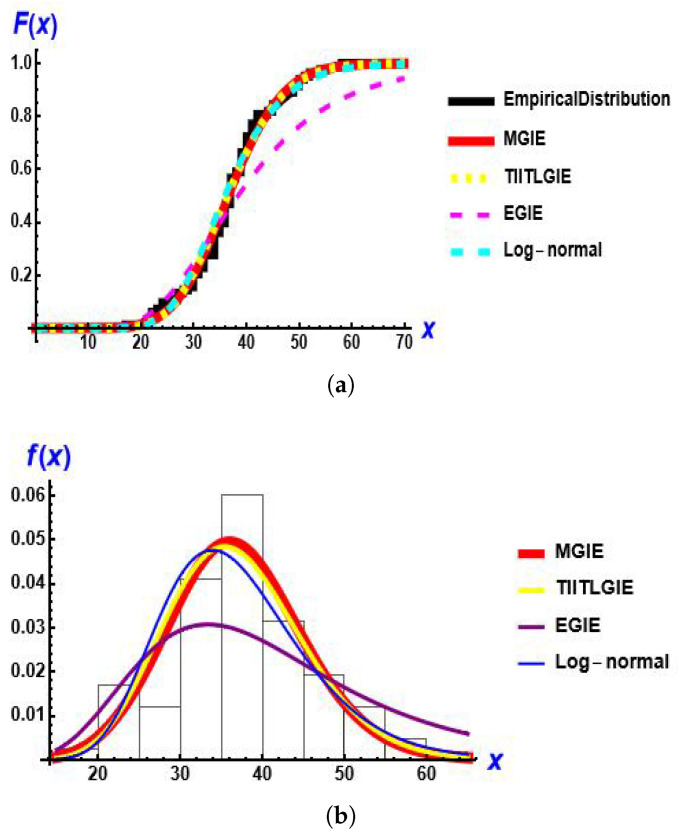
The fitted CDF and PDF Plots of the MGIE distribution compared to other distributions using data on the number of deaths due to COVID-19. (**a**) estimated CDF; (**b**) estimated PDF.

Figure 9 presents the empirical distribution, estimated CDF and estimated PDF for the models for this data set.

### 7.2. Data Set 2

This data shows the rainfall recorded in Los Angeles (30 June–1 July) in the period (1962–2012) [10]: 3.21, 4.42, 7.17, 7.22, 7.35, 7.66, 7.77, 7.93, 8.08, 8.11, 8.38, 8.69, 8.98, 9.08, 9.09, 9.24, 10.43, 10.71, 11.47, 11.57, 12.31, 12.32, 12.4, 12.46, 12.48, 12.82, 13.19, 13.53, 13.69, 14.35, 14.92, 16.36, 16.49, 16.58, 17.86, 17.94, 19.67, 20.2, 20.44, 21.0, 21.26, 22.0, 24.35, 26.98, 27.36, 27.47, 31.01, 31.25, 33.44, 37.25

We will calculate the descriptive statistics for this data as shown in the Table 7.

**Figure 10:**
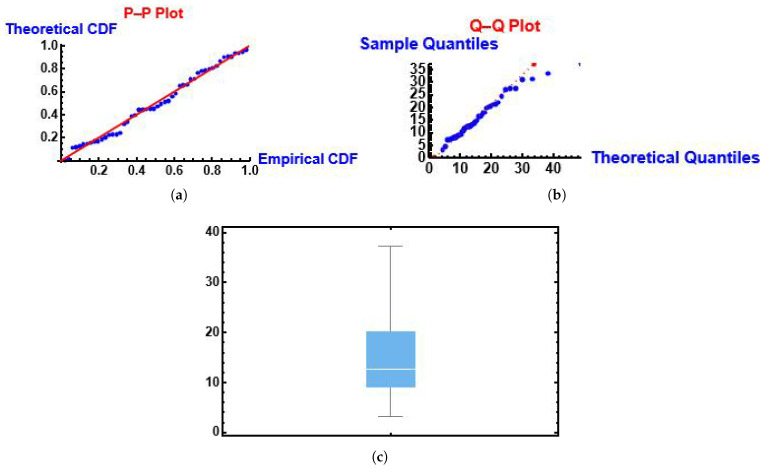
PP, QQ and box plots of the MGIE distribution using rainfall data. (**a**) PP plot; (**b**) QQ plot; (**c**) box plot.

From Figure 10 we can see that the MGIE distribution follows the fitted PP (probability-probability) and QQ (quantile-quantile) functions. Also, we can see the box plot.

Table 8 presents the Kolmogorov-Smirnov test to determine the goodness of fit and the *p*-value for MGIE distribution and competing models.

From Table 9 we note that the MGIE distribution consistently outperforms competitive distributions in all the criteria used for the study. This gives the MGIE distribution practically significant and supports its use as a flexible and effective alternative.

**Figure 11:**
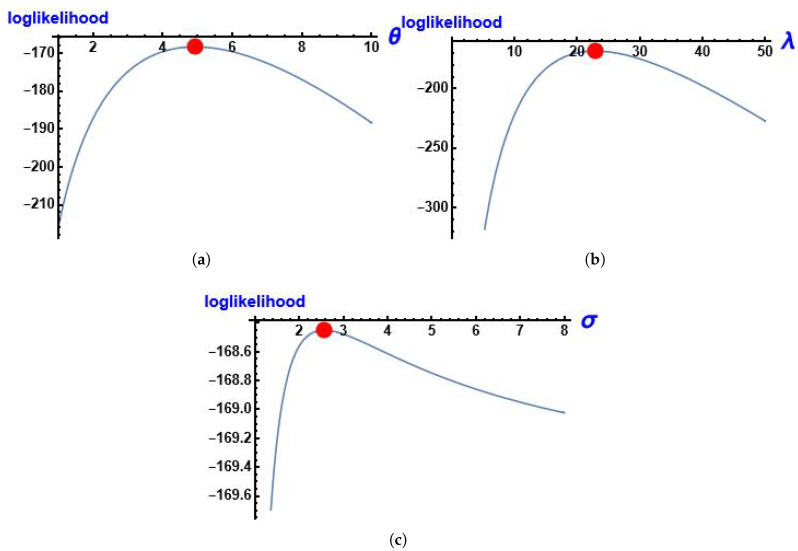
The profile of the log-likelihood function of the parameters using rainfall data. (**a**) log-likelihood function of θ; (**b**) log-likelihood function of λ; (**c**) log-likelihood function of σ.

Figure 11 shows the log-likelihood function of the parameters of MGIE distribution θ, λ and σ using this data.

**Figure 12:**
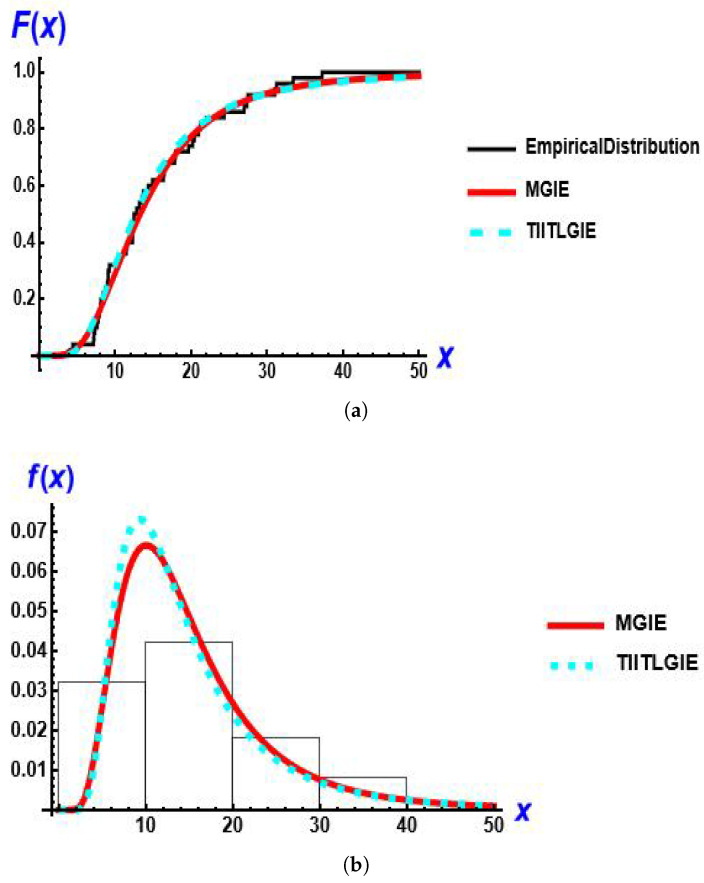
The fitted CDF and PDF plots of the MGIE distribution compared to other distributions using rainfall data. (**a**) estimated CDF; (**b**) estimated PDF.

Figure 12 presents the empirical distribution, estimated CDF and estimated PDF for the models for this data set.

### 7.3. Data Set 3

This data shows the lifespans of patients undergoing analgesic treatment [26]: 1.4, 1.1, 1.7, 1.3, 1.8, 1.9, 2.2, 1.6, 2.7, 1.7, 1.8, 4.1, 1.2, 1.5, 3, 1.4, 2.3, 1.7, 2.0, 1.6.

We will calculate the descriptive statistics for this data as shown in the Table 10.

**Figure 13:**
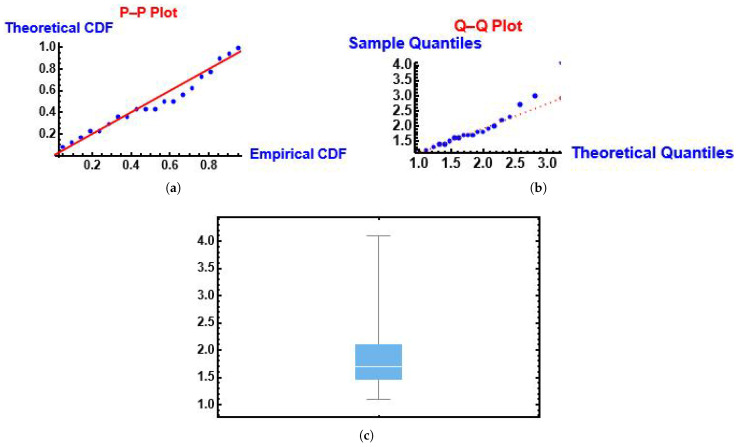
PP, QQ and box plots of the MGIE distribution using data of lifespans of patients undergoing analgesic treatment. (**a**) PP plot; (**b**) QQ plot; (**c**) box plot.

From Figure 13 we can see that the MGIE distribution follows the fitted PP (probability-probability) and QQ (quantile-quantile) functions. Also, we can see the box plot.

Table 11 presents the Kolmogorov-Smirnov test to determine the goodness of fit and the *p*-value for MGIE distribution and competing models.

From Table 12 we note that the MGIE distribution consistently outperforms competitive distributions in all the criteria used for the study. This gives the MGIE distribution practically significant and supports its use as a flexible and effective alternative.

**Figure 14:**
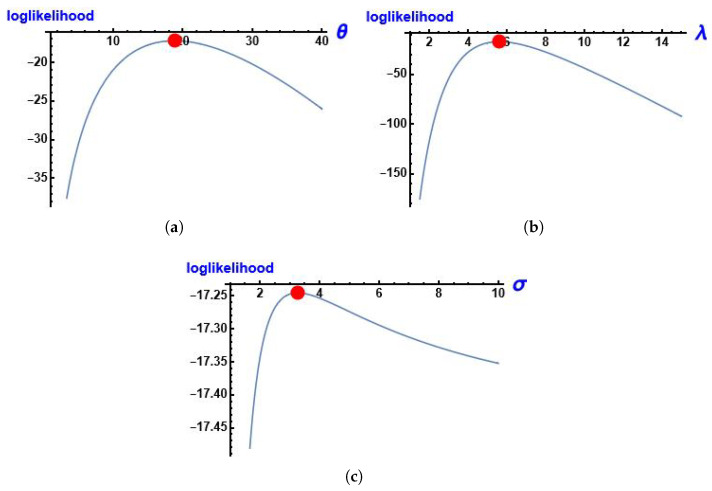
The profile of the log-likelihood function of the parameters using data on the lifespans of patients undergoing analgesic treatment. (**a**) log-likelihood function of θ; (**b**) log-likelihood function of λ; (**c**) log-likelihood function of σ.

Figure 14 shows the log-likelihood function of the parameters of MGIE distribution θ, λ and σ using this data.

**Figure 15:**
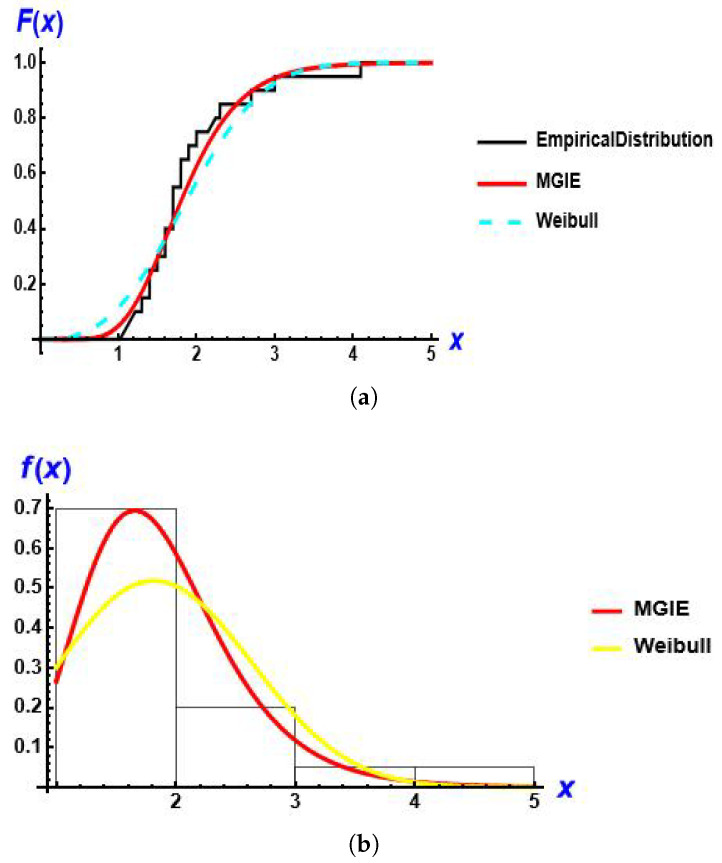
The fitted CDF and PDF plots of the MGIE distribution compared to other distributions using data of lifespans of patients undergoing analgesic treatment. (**a**) estimated CDF; (**b**) estimated PDF.

Figure 15 presents the empirical distribution, estimated CDF and estimated PDF for the models for this data set.

## 8. Conclusions

In this research, we presented a three-parameter Modified Generalized Inverted Exponential Distribution. Also, some important statistical properties are studied. The unknown parameters are estimated. In addition, we performed a simulation study to study the behavior of the estimators and present an application using real data from bio-sciences fields. As is clearly evident, our suggested distribution is more suitable than other related distributions.

Also, we present several propositions that can be helpful in future work, such as using the Bayesian methods to estimate the parameters of MGIED, estimating the three parameters of MGIED under several kinds of censored samples, and applying the model to different data areas in the field of bio-sciences data and in other fields.

## Figures and Tables

**Table 1:** The mean, median, mode, harmonic mean, skewness, and kurtosis of the MGIE distribution.

θ	λ	σ	Mean	Median	Mode	Harmonic Mean	Skewness	Kurtosis
2	2	1	4.18599	2.56696	1.49385	2.18182	0.320047	1.58616
2	2	1.5	3.71485	2.2405	1.26799	1.8	0.315947	1.58364
2	2	2	3.47929	2.07809	1.14125	1.65517	0.317213	1.58293
2	2	2.5	3.33795	1.98235	1.0698	1.57895	0.319189	1.58359
2	2	−2.5	2.20723	1.35201	0.809942	1.15385	0.31616	1.59818
2	2	−2	2.06589	1.29661	0.798997	1.11628	0.306128	1.58218
2	2	−1.5	1.83032	1.21543	0.784372	1.05882	0.28425	1.53551
2	2	−1	1.35919	1.08802	0.763937	0.96	0.230164	1.39708
2	2.5	2	4.34911	2.59761	1.42656	2.06897	0.317213	1.58293
2	3	2	5.21893	3.11713	1.71187	2.48276	0.317213	1.58293
2	3.5	2	6.08875	3.63665	1.99718	2.89655	0.317213	1.58293
2	4	2	6.95857	4.15617	2.28249	3.31034	0.317213	1.58293
2.5	2	2	2.53752	1.75299	1.07938	1.45044	0.278766	1.49894
3	2	2	2.06638	1.54675	1.02824	1.31148	0.250995	1.44738
3.5	2	2	1.78153	1.40311	0.985462	1.21014	0.229759	1.41264
4	2	2	1.58945	1.29661	0.949155	1.13246	0.21285	1.3877

**Table 2:** The MLE Estimates, bias, MSE and SE of MGIE for the unknown parameters $\hat{\theta }$, $\hat{\lambda }$ and $\hat{\sigma }$ with the initial values (θ = 0.2, λ = 0.7, σ = 1.5).

θ = 0.2, λ = 0.7, σ = 1.5					
*n*	Parameters	MLE	Bias	MSE	SE
10	$\hat{\theta }$	0.236909	0.0369095	0.00807538	0.00259226
	$\hat{\lambda }$	1.59447	0.894474	8.81476	0.0895695
	$\hat{\sigma }$	1.51459	0.0145933	0.443311	0.0210604
30	$\hat{\theta }$	0.210793	0.0107926	0.00132444	0.00109962
	$\hat{\lambda }$	0.817389	0.117389	0.331174	0.0178245
	$\hat{\sigma }$	1.57223	0.0722269	0.498665	0.0222248
50	$\hat{\theta }$	0.207648	0.00764813	0.000790327	0.000855901
	$\hat{\lambda }$	0.797576	0.0975759	0.210105	0.0141699
	$\hat{\sigma }$	1.59725	0.0972487	0.465888	0.0213749
75	$\hat{\theta }$	0.199697	−0.00030343	0.000778537	0.000882737
	$\hat{\lambda }$	0.753787	0.0537874	0.138475	0.0116498
	$\hat{\sigma }$	1.66174	0.161738	0.470234	0.0210836
100	$\hat{\theta }$	0.201508	0.00150826	0.000397691	0.000629136
	$\hat{\lambda }$	0.688347	−0.0116531	0.0974499	0.00986973
	$\hat{\sigma }$	1.55208	0.0520838	0.39711	0.0198694
150	$\hat{\theta }$	0.202435	0.00243459	0.000260734	0.000505036
	$\hat{\lambda }$	0.659425	−0.0405753	0.078573	0.00877517
	$\hat{\sigma }$	1.60264	0.102644	0.510432	0.0223696
200	$\hat{\theta }$	0.198672	−0.00132807	0.000208836	0.000455279
	$\hat{\lambda }$	0.709907	0.0099071	0.0495477	0.00703555
	$\hat{\sigma }$	1.73048	0.230478	0.412419	0.0189647

**Table 3:** The MLE Estimates, bias, MSE and SE of MGIE for the unknown parameters $\hat{\theta }$, $\hat{\lambda }$ and $\hat{\sigma }$ with the initial values (θ = 2, λ = 2, σ = 3).

θ = 2, λ = 2, σ = 3					
*n *	Parameters	MLE	Bias	MSE	SE
10	$\hat{\theta }$	2.91847	0.918466	5.02286	0.0646797
	$\hat{\lambda }$	2.26576	0.265758	1.0119	0.0306956
	$\hat{\sigma }$	2.51685	−0.483147	1.00199	0.0277367
30	$\hat{\theta }$	2.24674	0.246744	0.418058	0.0189085
	$\hat{\lambda }$	2.03333	0.0333324	0.217058	0.0147025
	$\hat{\sigma }$	2.57251	−0.427489	0.965469	0.0279912
50	$\hat{\theta }$	2.16094	0.160938	0.238289	0.0145808
	$\hat{\lambda }$	1.99578	−0.00422403	0.13916	0.0118018
	$\hat{\sigma }$	2.65198	−0.348019	0.948337	0.0287758
75	$\hat{\theta }$	2.07718	0.0771843	0.130341	0.0111583
	$\hat{\lambda }$	1.94481	−0.0551944	0.101414	0.00992298
	$\hat{\sigma }$	2.64109	−0.358909	0.945028	0.0285837
100	$\hat{\theta }$	2.66646	−0.33354	0.94224	0.0288413
	$\hat{\lambda }$	1.94512	−0.0548784	0.0834111	0.00897106
	$\hat{\sigma }$	1.55208	0.0520838	0.39711	0.0198694
150	$\hat{\theta }$	2.02861	0.0286072	0.0606204	0.00773705
	$\hat{\lambda }$	1.93442	−0.0655751	0.0627344	0.00764806
	$\hat{\sigma }$	2.71742	−0.282576	0.963623	0.0297432
200	$\hat{\theta }$	2.02628	0.0262832	0.0426176	0.00647833
	$\hat{\lambda }$	1.94182	−0.0581784	0.0493648	0.00678425
	$\hat{\sigma }$	2.71902	−0.280977	0.882471	0.0283607

**Table 4:** Descriptive statistics using the number of deaths due to COVID-19 data.

Max.	Min.	Mean	Median	Variance	SE	SD	Skewness	Kurtosis	Q1	Q3
58	17	36.9398	37	70.3988	0.920966	8.3904	0.090487	3.038	32	41

**Table 5:** The values of the Kolmogorov–Smirnov test and *p*-value of the fitted models using the data for the number of deaths due to COVID-19.

	MGIE	TIITLGIE	EGIE	Log-Normal
Kolmogorov-Smirnov	0.0940978	0.106065	0.211107	0.131228
*p*-value	0.42815	0.287172	0.0.00100567	0.104689

**Table 6:** Estimated parameters for some distributions using data on the number of deaths due to COVID-19.

Model	Parameters			LL	AIC	BIC	CAIC	HQIC
	$\hat{\theta }$	$\hat{\lambda }$	$\hat{\sigma }$					
MGIE	76.5019 SE (36.9912)	160.742 SE (23.9075)	2.04754 SE (1.45686)	−293.79	593.58	600.837	593.884	596.495
TIITLGIE	2.47119	95.9063	24.1311	−294.494	594.989	602.245	595.293	597.904
EGIE	10.9444	96.4891	1.37449	−309.413	624.826	632.082	625.13	627.741
log-normal	3.58195	0.239803	—	−296.555	597.11	601.948	597.26	599.054

**Table 7:** Descriptive statistics using rainfall data.

Max.	Min.	Mean	Median	Variance	SE	SD	Skewness	Kurtosis	Q1	Q3
37.25	3.21	15.1988	12.65	63.9671	1.13108	7.99795	0.954992	3.18019	8.98	20.2

**Table 8:** The values of the Kolmogorov–Smirnov test and *p*-value of the fitted models using rainfall data.

	MGIE	TIITLGIE
Kolmogorov–Smirnov	0.0802198	0.0818563
*p*-value	0.878882	0.863851

**Table 9:** Estimated parameters for some distributions using rainfall data.

Model	Parameters			LL	AIC	BIC	CAIC	HQIC
	$\hat{\theta }$	$\hat{\lambda }$	$\hat{\sigma }$					
MGIE	4.73508 SE (107.522)	23.2537 SE (1212.61)	2.48083 SE (775.005)	−168.453	342.906	348.642	343.427	345.09
TIITLGIE	2.51601	14.8464	1.50485	−169.152	344.304	350.04	344.825	346.488

**Table 10:** Descriptive statistics using data of the lifespans of patients undergoing analgesic treatment.

Max.	Min.	Mean	Median	Variance	SE	SD	Skewness	Kurtosis	Q1	Q3
4.1	1.1	1.9	1.7	0.495789	0.157447	0.704123	1.71975	5.92411	1.4	2.0

**Table 11:** The values of the Kolmogorov–Smirnov test and *p*-value of the fitted models using data on the lifespans of patients undergoing analgesic treatment.

	MGIE	Weibull Distribution
Kolmogorov-Smirnov	0.151588	0.18497
*p*-value	0.692487	0.447221

**Table 12:** Estimated parameters for some distributions using data of the lifespans of patients undergoing analgesic treatment.

Model	Parameters			LL	AIC	BIC	CAIC	HQIC
	$\hat{\theta }$	$\hat{\lambda }$	$\hat{\sigma }$					
MGIE	18.816 SE (26.4035)	5.61195 SE (0.953952)	3.26565 SE (4.49962)	−17.2453	40.4906	43.4778	41.9906	41.0737
weibull distribution	2.78703	2.12998	—	−20.5864	45.1728	47.1643	45.8787	45.5616

## Data Availability

The original contributions presented in this study are included in the article. Further inquiries can be directed to the corresponding authors.

## Abbreviations

The following abbreviations are used in this manuscript:

NModi-G	New modified-G (NModi-G) family
CDF	Cumulative Distribution Function
PDF	Probability Density Function
GIED	Generalized Inverted Exponential Distribution
TLGIE	Topp–Leone Inverted Exponential Distribution
TIITLGIE	Type II Topp-Leone Generalized Inverted Exponential Distribution
MGIE	Modified Generalized Inverted Exponential
G(y;σ,ϕ)	CDF of NModi-G family
g(y;σ,ϕ)	PDF of NModi-G family
F(x)	CDF of GIED
f(x)	PDF of GIED
G(x)	CDF of MGIE
g(x)	PDF of MGIE
MIE	Modified Inverse Exponential
MSIE	Modified Standard Inverse Exponential
MGSIE	Modified Generalized Standard Inverse Exponential
Q(u)	Quantile Function
m	Median
$\overset{´}{\mu_{r}}$	The $r^{th}$ Moment
$E_{r}\left(1\right)$	Generalized Exponential Integral Function
$M_{x}\left(t\right)$	Moment-Generating Function (MGF)
B	Bowley’s Coefficient of Skewness
M	Moors’ Coefficient of Kurtosis
R(x)	Reliability Function

h(x)	Hazard Function
O(x)	Odds Function
$H_{m}\left(x\right)$	Harmonic Mean
D(μ)	Mean Deviation
D(m)	Median Deviation
$g(x_{j})$	Order Statistics
$g(x_{n})$	Largest Order Statistic
$g(x_{1})$	Smallest Order Statistic
$R_{\delta }\left(x\right)$	Rényi entropy
L	Likelihood Function
l	Log-Likelihood Function
IE	Inverted Exponential Distribution
E	Exponential Distribution
EGIE	Exponentiated generalized inverted exponential distribution
AIC	Akaike Information Criterion
BIC	Bayesian Information Criterion
CAIC	Corrected Akaike Information Criteria
HQIC	Hannan–Quinn information criterion
MLE	likelihood estimate
MSE	Mean squared error
SE	Standard Error
SD	Standard deviation

## Appendix A

### Appendix A.1

**Proof of Theorem** **1.** Suppose the g(x) is the pdf of the MGIE distribution, then the Rényi entropy is derived as follow:

(A1) $$R_{\delta }\left(x\right)=\frac{1}{1-\delta }\left(log\;\left[J\left(\delta \right)\right]\right),$$

such that the:

$$J\left(\delta \right)=\int_{0}^{\infty }g^{\delta }\left(x\right)dx;\;\delta >0\;\mathrm{and}\;\delta \neq 1,$$

by using Equation (5) in the previous equation:

$$J\left(\delta \right)=\int_{0}^{\infty }{\left(\frac{\theta \lambda }{x^{2}}\right)}^{\delta }\;e^{\frac{-\lambda }{x}\delta }\;{\left(1-e^{\frac{-\lambda }{x}}\right)}^{(\theta -1)\delta }\;{\left(1+\frac{1}{\sigma }-\frac{2}{\sigma }{\left(1-e^{\frac{-\lambda }{x}}\right)}^{\theta }\right)}^{\delta }dx,$$

suppose

$$u=\frac{\lambda }{x},$$

therefore

$$J\left(\delta \right)=\int_{0}^{\infty }\lambda {\left(\frac{\theta u^{2}}{\lambda }\right)}^{\delta }\;e^{-u\delta }\;{\left(1-e^{-u}\right)}^{(\theta -1)\delta }\;{\left[1+\frac{1}{\sigma }-\frac{2}{\sigma }{\left(1-e^{-u}\right)}^{\theta }\right]}^{\delta }\;\frac{1}{u^{2}}\;du,$$

we derived the following equation by using binomial expansion

J(δ)=∫0∞λ(θu2λ)δe−uδ(1−e−u)(θ−1)δ×[∑i=0nδi(−1)i(2σ(1−e−u)θ)i(1+1σ)δ−i]1u2du,

J(δ)=λ(θλ)δ∑i=0nδi(−1)i(2σ)i(1+1σ)δ−i∫0∞e−uδ(1−e−u)(θ−1)δ+θiuδdu,

we derive the Rényi entropy for MGIE distribution as follow:

Rδ(x)=11−δ×log[λ(θλ)δ∑i=0n∑j=0nδi(θ−1)δ+θij(−1)i+j(2σ)i(1+1σ)δ−i(Γ(δ+1)(δ+j)δ+1)],

□

### Appendix A.2

(A2)
$$\frac{\partial l}{\partial \theta }=\frac{n}{\theta }+\sum_{i=1}^{n}\log\left(1-e^{\frac{-\lambda }{x_{i}}}\right)-\frac{2}{\sigma }\sum_{i=1}^{n}\frac{{\left(1-e^{\frac{-\lambda }{x_{i}}}\right)}^{\theta }\log\left(1-e^{\frac{-\lambda }{x_{i}}}\right)}{1+\frac{1}{\sigma }-\frac{2}{\sigma }{\left(1-e^{\frac{-\lambda }{x_{i}}}\right)}^{\theta }}=0,$$

(A3)
$$\frac{\partial l}{\partial \lambda }=\frac{n}{\lambda }-\sum_{i=1}^{n}\frac{1}{x_{i}}+\left(\theta -1\right)\sum_{i=1}^{n}\frac{e^{\frac{-\lambda }{x_{i}}}}{x_{i}\left(1-e^{\frac{-\lambda }{x_{i}}}\right)}-\frac{2\theta }{\sigma }\sum_{i=1}^{n}\frac{e^{\frac{-\lambda }{x_{i}}}{\left(1-e^{\frac{-\lambda }{x_{i}}}\right)}^{\theta -1}}{x_{i}\left(1+\frac{1}{\sigma }-\frac{2}{\sigma }{\left(1-e^{\frac{-\lambda }{x_{i}}}\right)}^{\theta }\right)}=0,$$

(A4)
$$\frac{\partial l}{\partial \sigma }=\sum_{i=1}^{n}\frac{-\frac{1}{\sigma 2}+\frac{2}{\sigma^{2}}{\left(1-e^{\frac{-\lambda }{x_{i}}}\right)}^{\theta }}{1+\frac{1}{\sigma }-\frac{2}{\sigma }{\left(1-e^{\frac{-\lambda }{x_{i}}}\right)}^{\theta }}=0.$$

Mathematica (13.0) will be used to find the maximum likelihood estimates of our parameters $\hat{\theta }$, $\hat{\lambda }$ and $\hat{\sigma }$ numerically.

### Appendix A.3

(A5)
$$\frac{\partial^{2}logl}{\partial \theta^{2}}=\frac{-n}{\theta^{2}}-\frac{2}{\sigma }\sum_{i=1}^{n}\left[\frac{{\left(1-e^{\frac{-\lambda }{x_{i}}}\right)}^{\theta }log{\left(1-e^{\frac{-\lambda }{x_{i}}}\right)}^{2}}{1+\frac{1}{\sigma }-\frac{2}{\sigma }{\left(1-e^{\frac{-\lambda }{x_{i}}}\right)}^{\theta }}-\frac{\frac{-2}{\sigma }{\left(1-e^{\frac{-\lambda }{x_{i}}}\right)}^{2\theta }log{\left(1-e^{\frac{-\lambda }{x_{i}}}\right)}^{2}}{{\left(1+\frac{1}{\sigma }-\frac{2}{\sigma }{\left(1-e^{\frac{-\lambda }{x_{i}}}\right)}^{\theta }\right)}^{2}}\right],$$

(A6)
$$\begin{array}{cc} \frac{\partial^{2}logl}{\partial \lambda^{2}} & =\frac{-n}{\lambda^{2}}+\left(\theta -1\right)\sum_{i=1}^{n}\left[\frac{-e^{\frac{-\lambda }{x_{i}}}}{{x_{i}}^{2}\left(1-e^{\frac{-\lambda }{x_{i}}}\right)}-\frac{e^{\frac{-2\lambda }{x_{i}}}}{{x_{i}}^{2}{\left(1-e^{\frac{-\lambda }{x_{i}}}\right)}^{2}}\right]\\ \; & -\frac{2\theta }{\sigma }\sum_{i=1}^{n}[\frac{e^{\frac{-\lambda }{x_{i}}}\left(\left(\theta -1\right)e^{\frac{-\lambda }{x_{i}}}{\left(1-e^{\frac{-\lambda }{x_{i}}}\right)}^{\theta -2}-{\left(1-e^{\frac{-\lambda }{x_{i}}}\right)}^{\theta -1}\right)}{{x_{i}}^{2}\left(1+\frac{1}{\sigma }-\frac{2}{\sigma }{\left(1-e^{\frac{-\lambda }{x_{i}}}\right)}^{\theta }\right)}\\ \; & -\frac{\frac{-2\theta }{\sigma }e^{\frac{-2\lambda }{x_{i}}}{\left(1-e^{\frac{-\lambda }{x_{i}}}\right)}^{2\theta -2}}{{x_{i}}^{2}{\left(1+\frac{1}{\sigma }-\frac{2}{\sigma }{\left(1-e^{\frac{-\lambda }{x_{i}}}\right)}^{\theta }\right)}^{2}}], \end{array}$$

(A7)
$$\frac{\partial^{2}logl}{\partial \sigma^{2}}=\sum_{i=1}^{n}\left[\frac{\frac{2}{\sigma^{3}}-\frac{4}{\sigma^{3}}{\left(1-e^{\frac{-\lambda }{x_{i}}}\right)}^{\theta }}{1+\frac{1}{\sigma }-\frac{2}{\sigma }{\left(1-e^{\frac{-\lambda }{x_{i}}}\right)}^{\theta }}-\frac{{\left(\frac{-1}{\sigma^{2}}+\frac{2}{\sigma^{2}}{\left(1-e^{\frac{-\lambda }{x_{i}}}\right)}^{\theta }\right)}^{2}}{{\left(1+\frac{1}{\sigma }-\frac{2}{\sigma }{\left(1-e^{\frac{-\lambda }{x_{i}}}\right)}^{\theta }\right)}^{2}}\right],$$

(A8)
$$\begin{array}{cc} \frac{\partial^{2}logl}{\partial \theta \partial \lambda } & =\sum_{i=1}^{n}\left[\frac{e^{\frac{-\lambda }{x_{i}}}}{x_{i}\left(1-e^{\frac{-\lambda }{x_{i}}}\right)}\right]-\frac{2}{\sigma }\sum_{i=1}^{n}[\frac{{\left(1-e^{\frac{-\lambda }{x_{i}}}\right)}^{\theta -1}e^{\frac{-\lambda }{x_{i}}}\left(\theta log\left(1-e^{\frac{-\lambda }{x_{i}}}\right)+1\right)}{x_{i}\left(1+\frac{1}{\sigma }-\frac{2}{\sigma }{\left(1-e^{\frac{-\lambda }{x_{i}}}\right)}^{\theta }\right)}\\ \; & -\frac{\frac{-2\theta }{\sigma }e^{\frac{-\lambda }{x_{i}}}{\left(1-e^{\frac{-\lambda }{x_{i}}}\right)}^{2\theta -1}log\left(1-e^{\frac{-\lambda }{x_{i}}}\right)}{x_{i}{\left(1+\frac{1}{\sigma }-\frac{2}{\sigma }{\left(1-e^{\frac{-\lambda }{x_{i}}}\right)}^{\theta }\right)}^{2}}], \end{array}$$

(A9)
$$\begin{array}{cc} \frac{\partial^{2}logl}{\partial \sigma \partial \theta } & =\sum_{i=1}^{n}[\frac{\frac{2}{\sigma^{2}}{\left(1-e^{\frac{-\lambda }{x_{i}}}\right)}^{\theta }log\left(1-e^{\frac{-\lambda }{x_{i}}}\right)}{\left(1+\frac{1}{\sigma }-\frac{2}{\sigma }{\left(1-e^{\frac{-\lambda }{x_{i}}}\right)}^{\theta }\right)}\\ \; & -\frac{\left(\frac{-1}{\sigma^{2}}+\frac{2}{\sigma^{2}}{\left(1-e^{\frac{-\lambda }{x_{i}}}\right)}^{\theta }\right)\left(\frac{-2}{\sigma }{\left(1-e^{\frac{-\lambda }{x_{i}}}\right)}^{\theta }log(1-e^{\frac{-\lambda }{x_{i}}}\right))}{{\left(1+\frac{1}{\sigma }-\frac{2}{\sigma }{\left(1-e^{\frac{-\lambda }{x_{i}}}\right)}^{\theta }\right)}^{2}}], \end{array}$$

(A10)
$$\frac{\partial^{2}logl}{\partial \sigma \partial \lambda }=\sum_{i=1}^{n}\left[\frac{\frac{2\theta }{\sigma^{2}}{\left(1-e^{\frac{-\lambda }{x_{i}}}\right)}^{\theta -1}e^{\frac{-\lambda }{x_{i}}}}{x_{i}\left(1+\frac{1}{\sigma }-\frac{2}{\sigma }{\left(1-e^{\frac{-\lambda }{x_{i}}}\right)}^{\theta }\right)}-\frac{\left(\frac{-1}{\sigma^{2}}+\frac{2}{\sigma^{2}}\left(1-e^{\frac{-\lambda }{x_{i}}}\right)\right)\left(\frac{-2\theta }{\sigma }{\left(1-e^{\frac{-\lambda }{x_{i}}}\right)}^{\theta -1}e^{\frac{-\lambda }{x_{i}}}\right)}{x_{i}{\left(1+\frac{1}{\sigma }-\frac{2}{\sigma }{\left(1-e^{\frac{-\lambda }{x_{i}}}\right)}^{\theta }\right)}^{2}}\right].$$
